# Intermittent Fasting and Hormonal Regulation: Pathways to Improved Metabolic Health

**DOI:** 10.1002/fsn3.70586

**Published:** 2025-08-07

**Authors:** Wijdan Shkorfu, Abdulmannan Fadel, Mohammed Hamsho, Yazan Ranneh, Hafiz Muhammad Shahbaz

**Affiliations:** ^1^ Department of Nutrition & Dietetics Faculty of Health Sciences, Bahçeşehir University Istanbul Turkey; ^2^ Department of Nutrition and Health College of Medicine and Health Sciences Al‐Ain UAE; ^3^ Department of Nutrition & Dietetics Faculty of Health Sciences, Istanbul Yeni Yuz Yil University Istanbul Turkey; ^4^ Department of Nutrition and Dietetics, College of Pharmacy Al Ain University Al‐Ain UAE; ^5^ Department of Nutrition and Health, College of Medicine and Health Sciences United Arab Emirates University Al‐Ain UAE

**Keywords:** cancer risk, circadian rhythm, hormonal systems, intermittent fasting, metabolic disorders

## Abstract

Intermittent fasting (IF), a temporal dietary pattern, has garnered interest in improving anthropometric and metabolic markers. Beyond this, IF appears to recalibrate hormonal circadian rhythms and reshape gut microbiota—two key intermediaries through which IF exerts effects on endocrine, inflammatory, and oncogenic pathways. This review synthesizes research findings of IF on key endocrine systems and outlines its potential implications for oncogenic risk. We primarily examine the effects of IF on hormonal regulation with a particular focus on its relevance to metabolic and oncogenic health outcomes. We explored hormonal alterations induced by various IF protocols and discussed their physiological implications. Controlled observations or interventional studies in both human and animal models were included. Evidence indicates that IF exerts systemic effects on hormonal rhythmicity, including insulin, thyroid hormones, glucocorticoids, and sex hormones, potentially re‐establishing homeostatic endocrine function. Moreover, IF influences cancer‐related pathways via modulation of endocrine axes and attenuation of inflammatory markers. These mechanisms offer a theoretical basis for IF's potential in attenuating metabolic dysfunction and cancer risk. However, the current research is limited by variations in study designs, short durations, limited cohorts, and population‐specific findings, restricting generalizability and applicability. Ultimately, IF represents a multifaceted dietary strategy with the potential to synchronize circadian and hormonal systems, positioning it as a promising intervention in metabolic and endocrine‐related conditions. However, whether long‐term IF can modulate specific hormonal axes without overt physiological side effects, including sex‐specific effects, remains unclear. To establish its clinical relevance and therapeutic safety, validation through well‐designed and large‐scale human trials is imperative.

## Graphical abstract

Intermittent fasting regulates key hormonal pathways affecting metabolic health. Intermittent fasting improves insulin sensitivity and β‐cell response. Intermittent fasting preserves circadian rhythm by advancing melatonin secretion.

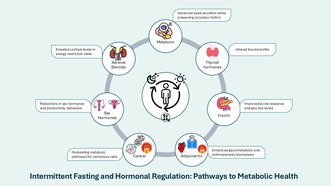

## 1 Introduction

The rapid technological advancements have significantly altered human eating and sleeping patterns, which have profound implications for circadian rhythms and metabolic health. The ability to work, eat, and remain active throughout the night represents a departure from traditional practices observed by our early ancestors. The human body naturally experiences rhythmic fluctuations in various biological functions, including cellular processes, physiological responses, and behavioral patterns, all following a 24‐h cycle (Fatima and Rana 2020). For example, heart rate, blood pressure, insulin sensitivity, and body temperature tend to rise in the morning and decline in the evening (Fatima and Rana 2020; Carrasco‐Benso et al. 2016). These cyclic changes, known as circadian rhythms, are regulated by an internal timing system called the circadian clock, which operates on approximately 24‐h cycles (Fatima and Rana 2020).

In living organisms, the circadian clock synchronizes oscillations in cellular activities, biochemical reactions, physiological functions, and even behaviors with the changing solar cycles throughout the seasons (Fatima and Rana 2020). Although the central clock that governs circadian rhythms is located in the suprachiasmatic nucleus in the brain, various peripheral clocks reside in different tissues and organs of the body (Fatima and Rana 2020). These peripheral clocks rely on external cues—known as zeitgebers or “time givers”—to align our internal clock with the external environment. Circadian rhythms regulate key biological functions, including the sleep–wake cycle, body temperature, blood pressure, hormone secretion, and metabolism (Fatima and Rana 2020).

Chronic perturbation of physiological rhythms—particularly through irregular schedules or shift‐based occupations—significantly amplifies the probability of age‐related pathologies and compromises systemic homeostasis (Manoogian and Panda 2016). The genomic apparatus and tissue‐specific oscillatory mechanisms exhibit temporal sensitivity to both nutritional input and diurnal variations. Contemporary lifestyle patterns, often characterized by extended periods of caloric consumption, frequently result in excessive energy intake, which in turn influences the synchronization of organ‐specific circadian regulators (Manoogian and Panda 2016). Research has consistently linked circadian rhythm disruptions to metabolic dysregulation, increasing susceptibility to cardiometabolic disorders such as obesity, diabetes, metabolic syndrome, and cardiovascular diseases (Fatima and Rana 2020). Animal and human studies further confirm the adverse metabolic effects of circadian disruption (Flanagan et al. 2020; Meléndez‐Fernández et al. 2023), highlighting its influence on hormonal and systemic balance.

Historically, the dietary patterns of our ancestors closely resembled intermittent fasting (IF), which naturally aligned with circadian rhythm. This dietary model involved fasting during food scarcity and eating when available. Fasting is believed to be an adaptation response that enhances survival during harsh conditions like famine. Beyond its evolutionary roots, IF has been practiced for centuries as a spiritual or religious observance. Today, IF has gained prominence for its potential role in weight management and overall well‐being by strategically adjusting meal timing. Aligning meals with the body's internal clock and peak metabolic activity not only impacts metabolic efficiency but also plays a key role in regulating hormonal signals associated with appetite, energy metabolism, and homeostasis. IF and time‐restricted eating provide strategies to improve this alignment, which will help prevent or mitigate metabolic disorders and enhance health outcomes (Wehrens et al. 2017; Flanagan et al. 2020; Charlot et al. 2021; Smith and Betts 2022). The profound changes brought about by technology, the complex interplay between the circadian clock and metabolism, and the historical and contemporary significance of IF are captivating avenues for study with implications for human health and well‐being. Within this framework, the purpose of this review is to explore how different hormonal systems and essential physiological processes are affected by the modern practice of IF.

### 1.1 IF and Health Benefits

IF, characterized by distinct periods of fasting and feeding, has been rising in popularity, given its link to several short‐ and long‐term health advantages (Mandal et al. 2022). During the fasting window, caloric intake is abstained, whereas during the feeding window, all caloric intake is consolidated (Mandal et al. 2022). Among the various forms of IF, three main types have been the subject of research, namely time‐restricted feeding (TRF), alternate‐day fasting (ADF), and 5:2 fasting (Varady 2016). Irrespective of the specific approach, studies have postulated that fasting provides multiple benefits for human health, including weight and fat mass loss, improvements in cognitive function and cardiovascular health, inflammation, metabolic syndrome, insulin resistance, and potential roles in cancer prevention and longevity (Meléndez‐Fernández et al. 2023).

Two variations of ADF have been utilized: the zero‐calorie model, which permits only water consumption, and a modified approach, which allows only 25% of caloric intake, with ad libitum eating permitted on feasting days. Similarly, the 5:2 fast involves restricting caloric intake to 500–1000 kcal/day for two consecutive or nonconsecutive fasting days while consuming a normal diet on the remaining 5 days (Varady 2016).

### 1.2 Mechanisms for IF

Several theories have been proposed to explain the mechanisms underlying IF. According to the ketogenesis theory, IF induces an adaptive metabolic shift in response to limited energy and relatively low glucose levels, leading to the preferential production of ketone bodies for energy. Furthermore, the theory of circadian rhythms proposes that aligning eating patterns with the body's internal clock improves metabolic health by influencing hormone secretion and response, whereas a misalignment worsens the regulation of glycemia, lipids, and blood pressure by increasing pro‐inflammatory markers. Moreover, the oxidative stress theory maintains that the energy restriction associated with IF reduces the production of reactive oxygen species, thereby minimizing cellular damage and promoting longevity (Dong et al. 2020; Vasim et al. 2022). These mechanisms collectively shape fasting‐induced metabolic transitions from glucose to lipid utilization, mostly through ketone body production from the liver after 12–36 h of food deprivation (Mattson et al. 2018; Mattson 2025; Hofer et al. 2021). This shift is influenced by glycogen reserves and physical activity levels and stabilizes over subsequent days. Reduced nutrient availability during fasting triggers crucial metabolic pathways regulated by mTOR, sirtuins, and AMPK (Mattson et al. 2018; Mattson 2025; Hofer et al. 2021). While diminished glucose and amino acid levels deactivate mTOR, reducing protein synthesis and triggering autophagy—an intracellular recycling process crucial for eliminating damaged organelles and proteins that also diminish with aging (Mattson et al. 2018; Mattson 2025; Hofer et al. 2021). AMPK is activated during low‐energy states, resulting in increased ketogenesis and fatty acid oxidation. Moreover, the decrease in insulin and IGF‐1 signaling triggers hormetic cellular stress responses as a result of nutrient‐deprivation adaptations, leading to upregulation of protective mechanisms by improved organ function, mitigating age‐related decline, and alleviating inflammation, all of which may enhance extended healthspan (Mattson et al. 2018; Mattson 2025; Hofer et al. 2021). In contrast to the fed state, where a reversible metabolic shift promotes cellular growth and recovery (Mattson et al. 2018; Hofer et al. 2021; De Cabo and Mattson 2019). Additionally, IF‐induced metabolic shifts influence circadian rhythms, gut microbiota composition, and hormonal balance (Daas and De Roos 2021; Haupt et al. 2021).

However, the advantages of IF may also be explained by its impact on the balance of gut flora. IF induces consistent shifts in gut flora compositions and diversity, notably enriching metabolically favorable SCFA‐producing taxa while reducing pathogenic ones. Thus, improved SCFA production and improving gut barrier function, metabolic markers, and attenuated inflammation (Pieczyńska‐Zając et al. 2023; Su et al. 2021; Li et al. 2017). According to research, this eating pattern contributes to the microbial ecosystem's increased variety, which is linked to better glycemic management and decreased inflammation (Angoorani et al. 2021). Furthermore, research on animals has demonstrated that these microbial alterations could promote the conversion of white fat to brown fat, which, in turn, supports efforts to address obesity (Li et al. 2017). Studies on Ramadan fasting and time‐restricted eating have shown similar trends, with gut microbiome remodeling and blooming in beneficial SCFA‐producing bacteria (Pieczyńska‐Zając et al. 2023; Su et al. 2021). Consequently, a major contributing aspect of the explanation of many health advantages of IF is the regulation of gut microbiota through metabolic and immunoregulatory effects (Pieczyńska‐Zając et al. 2023; Su et al. 2021; Li et al. 2017). Irrespective of the discussed theories concerning IF, it is evident that each contributes to the regulation of the endocrine system.

### 1.3 Hormonal Regulation and Fasting

The intricate control over the circadian rhythms of endogenous metabolic and hormonal processes involves a complex network of central and peripheral pacemakers (Kuula et al. 2022), which can be influenced by various external factors, such as the sleep–wake cycle, light–dark cycles, nutritional patterns, and timing of physical activity. As such, our comprehensive review aims to summarize the impact of fasting and time‐restricted eating (TRE) on hormonal responses. Considering the current lack of reviews specifically examining the effects of fasting regimens on a range of hormones, our review fills a notable gap in the existing literature. Despite the limited number of existing reviews assessing the effects of fasting on certain hormones, the current review thoroughly analyzes the effects of IF on hormones and the endocrine system in both animal and human models.

By synthesizing available evidence, we aim to provide valuable insights into the intricate relationship between fasting, hormonal responses, and regulating circadian rhythms, shedding light on the potential implications of IF for human health and well‐being. This review focuses on the effects of IF on various endocrine systems, including insulin regulation, thyroid function, adrenal steroid production, and reproductive hormone balance. Additionally, we examine fasting's potential contributions to cancer prevention, particularly its impact on metabolic and hormonal pathways.

## 2 Methodology

A comprehensive literature search was conducted using the Google Scholar, PubMed, and Semantic Scholar databases to identify studies published up to January 31, 2024, without any time restrictions. A subsequent search was then conducted to include the most recent publications up to August 17, 2024. The search was repeated on May 25, 2025, to include any recent publications. The search focused on animal studies, human observational studies, and randomized controlled trials (RCTs). The key search terms included “intermittent fasting,” “hormonal response,” “circadian rhythm,” and “metabolic health.” Studies were included based on their relevance to the topic, and those not focusing on hormonal or metabolic outcomes were excluded.

## 3 Insulin Regulation and Glucose Metabolism

### 3.1 Insulin Sensitivity and Secretion

Insulin, a hormone synthesized by the pancreatic beta‐cells, plays a central role in glucose homeostasis by facilitating cellular glucose uptake for immediate energy utilization or storage. Besides insulin production and release, insulin sensitivity also displays specific circadian patterns and is influenced by molecular clocks within insulin‐sensitive organs (Kim et al. 2021). Emerging evidence suggests that IF influences the regulation of insulin across both animal and human subjects. Table 1 presents a comparative summary of insulin responses observed in both models.

**TABLE 1:** Hormonal and metabolic effects of intermittent fasting.

Hormone category	Fasting type	Type of participants		Principal findings	References
		Human models	Animal models		
Insulin regulation	5:2 fast	Humans	/	↓ 50% in insulin, ↓ HOMA‐IR, ↓ Body weight	Harvie et al. (2011), Xiaoyu, Yuxin, and Li (2024), Silva et al. (2023)
		T2DM		↑ Risk of hypoglycemia	Corley et al. (2018)
	ADF	Humans	Mice	↓ Insulin in animal models, ↓ 50% in insulin was seen in humans	Duan et al. (2003), Anson et al. (2003), Hoddy et al. (2020), Heilbronn et al. (2004), Cho et al. (2019)
	TRF	Humans	Animals	↑ Insulin sensitivity, ↑ Gut microbial diversity, ↓ fasting plasma glucose, ↓ Body weight, ↓ Inflammation, ↓ Dyslipidemia, ↑ HDL‐C, ↑ Browning of iWAT, ↑ Uncoupling protein	Xie et al. (2022), Aouichat et al. (2020)
	eTRF	Prediabetes	/	↑ β‐cell response to glucose	Sutton et al. (2018)
	IF	w/out DM 1 and 2	/	↑ Metabolic parameters, ↑ Anthropometry, ↑ C‐peptide and adiponectin, ↓ Leptin and Hepatic fat content	Yuan et al. (2022), Albosta and Bakke (2021), Muñoz‐Hernández et al. (2020), Song and Kim (2023), Haupt et al. (2021), Xiaoyu, Yuxin, and Li (2024)
		T1DM	/	↓ Risk of hypoglycemia, ↓ Glycemic variability, ↑ Lipid metabolism, ↓ Endogenous glucose production, ↑ Insulin sensitivity, ↓ Body weight, ↑ Body composition	Herz et al. (2023)
Thyroid hormones	Fasting	Humans	Rats	(−) fasting fT4, (−) prolonged fasting fT4, ↓ TSH, ↓ T4, ↓ T3	Mahadevan et al. (2017), Nair et al. (2014), de Vries et al. (2014)
	24‐h fast	Humans	/	↓ by 55% in serum T3, (−) TSH	Merimee et al. (1976)

	ADF	Humans	Rats	↓ Circulating serum T3, (−) TSH	Stekovic et al. (2019), García‐Luna et al. (2023)
		Subclinical hypothyroidism and obesity	/	↓ Body weight, ↓ Body fat	Akasheh et al. (2019)
	Ramadan/Islamic fasting	Dysfunctional thyroid and taking levothyroxine	/	↓ fT4, ↓ fT3, ↑ TSH	Barati et al. (2023)
		Humans		↓ T3, ↑ rT3, (↓ T4, ↓ T3 in last days of Ramadan fasting in women)	Azizi et al. (2015)
IF	Hypothyroidism	/	↓ fT3, ↓ fT4, ↓ TSH	Belal et al. (2023)
Adrenal cortical steroids	24‐h fast	Obese adults	/	↑ Cortisol	Marciniak et al. (2023)
	Prolonged fast (72‐h)	Humans	/	↑ Cortisol, ↑ ACTH, ↑ Adrenaline, ↑ Noradrenaline, ↓ TSH, ↓ T3	Beer et al. (1989)
	Fasting	Healthy volunteers	/	↑ Cortisol, ↑ Cortisol half‐life, ↑ Secretory activity, ↑ Number of secretory episodes, ↓ Weight	Fitcher et al. (1986)
		Humans	/	↓ T3, ↓ T4, ↓ TSH, ↑ rT3, ↑ Growth hormone, ↑ Cortisol, ↑ Adrenaline	Palmblad et al. (1977)
		Hospitalized	/	↓ Body weight, ↑ Cortisol, ↑ DHEAs, ↑ NK cell activity, ↓ CD4	Komaki et al. (1997)
		Lean and Obese	/	↑ DHEA, ↑ ACTH, ↑ Cortisol	Korbonits et al. (1996)
		Healthy	/	↓ Prolactin, ↓ T3, ↑ Cortisol, ↑ DHEAs, ↑ SHBG, ↑ Albumin, ↓ free testosterone	Tegelman et al. (1986)
	Short‐term	Young vs. old	/	↑ Cortisol	Bergendahl et al. (2000)
	TRF	Humans	/	↓ Cortisol in groups without CR and ↑ Cortisol in isocaloric group	Chawla et al. (2021)
Reproductive hormones	Fasting	/	Rabbits	↓ LH peak, ↓ Estradiol‐17β, ↓ Receptivity and fertility rates	Brecchia et al. (2006)
			Ewes	↓ Insulin, ↓ IGF‐1, ↑ Progesterone	Kiyma et al. (2004)
		Humans	/	↑ Urinary Gonadotropin, ↓ Testosterone, (−) FSH, (−) LH	Kyung et al. (1985)
	Short‐term Fasting	/	Zebra finches	↑ Corticosterone, ↓ Testosterone, ↓ Reproductive behavior	Lynn et al. (2010)
		Humans	/	↓ LH, ↓ FSH, ↓ Testosterone, ↓ free testosterone, ↓ Weight	Klibanski et al. (1981)
		Humans		(−) LH, (−) GH, ↑ GH secretion rate, ↑ Cortisol, (−) Estradiol, (−) Progesterone, ↓ Leptin, ↓ IGF‐1, ↓ Insulin	Bergendahl et al. (1999)
	IF	/	Young rats	↓ Body weight, ↓ Blood glucose, ↓ Ovarian weight, ↓ LH, ↑ Estradiol, ↓ Testosterone, ↓ Leptin, ↓ GnRH	Kumar et al. (2013)
	Winter fast	/	*Salvelinus alpinus*	↓ Mass, ↓ Fecundity, ↓ Oocyte recruitment, ↓ Temporal pattern of plasma E2, ↓ GH, ↓ IGF‐1	Frantzen et al. (2004)
	TRF	/	Animal	(−) Minimal impact on estradiol,↓ Testosterone, ↓ IGF‐1, ↓ T3, ↑ Adiponectin	Hua et al. (2020), Moro et al. (2021), Stratton et al. (2020), Moro et al. (2016), Moro et al. (2020)
	eTRF	Humans	/	↓ FAI, ↑ SHBG, ↓ weight, ↓Testosterone, ↓ IGF‐1, ↑ eumenorrhea and fertility	Cienfuegos et al. (2022), Moro et al. (2016), Moro et al. (2020), Stratton et al. (2020), Moro et al. (2021), Li et al. (2021) Harvie et al. (2010), Jakubowicz et al. (2013), Velissarious et al. (2025)

	48‐h Fast	Humans	/	↓ FSH, ↓ LH, ↓ frequency of pulsatile LH secretion, ↓ insulin, ↑ β‐hydroxybutyrate	Cameron et al. (1991)
		Humans	/	↓17‐β‐estradiol in females, ↑ Norepinephrine in females, ↑ Psychological stress	Solianik et al. (2023)
	72‐h Fast	Humans	/	Altered LH secretory dynamics, ↑ GH, ↓ IGF‐1, ↓ T3, ↓ TSH	Olson et al. (1995)
		Humans	/	↑ β‐hydroxy‐butyric acid, ↓ Free thyronine, (−) LH pulsatility, FSH, estradiol, progesterone, ↑ Cortisol, ↑ Advance in melatonin secretion	Berga et al. (2001)
Islamic/Ramadan Fast	Humans	/	(−) FSH, (−) LH, (−) Testosterone, (−) Prolactin, (−) TSH, (−) T3, ↑ T4, ↑ fT4 Index, ↓ Weight	Azizi (1991)

Abbreviations: −, no change; ACTH, adrenocorticotropic hormone; ADF, alternate‐day fasting; CD4, cluster of differentiation 4; CR, calorie restriction; DHEAs, dehydroepiandrosterone sulfate; eTRF, early time‐restricted feeding; FAI, free androgen index; FSH, follicle‐stimulating hormone; fT3, free triiodothyronine; fT4, free thyroxine; GH, growth hormone; GnRH, gonadotropin‐releasing hormone; HOMA‐IR, homeostatic model of insulin resistance; IF, intermittent fasting; IGF‐1, insulin‐like growth factor 1; iWAT, inguinal white adipose tissue; LH, luteinizing hormone; NK cells, natural killer cells; SHBG, sex hormone‐binding globulin; T1DM, Type 1 diabetes mellitus; T2DM, Type 2 diabetes mellitus; TRF, time‐restricted feeding; TSH, thyroid‐stimulating hormone.

Multiple studies in rodent models consistently demonstrate that IF leads to significant reductions in insulin levels compared to ad libitum feeding (Duan et al. 2003). Moreover, investigations into ADF in mice yielded contrasting conclusions (Anson et al. 2003; Hoddy et al. 2020). One study attributed decreased insulin levels to reduced food intake (Anson et al. 2003), whereas another suggested that caloric intake or the quantity of food consumed had no effect on the decrease in insulin levels (Hoddy et al. 2020). More recent analyses propose that reductions in insulin levels could be ascribed to adaptive modifications in pancreatic beta‐cells’ transcriptional regulation of the insulin gene in response to fasting conditions (Karimi et al. 2024). Human research, on the other hand, associates reduction in insulin levels with progressive decrease in anthropometric parameters and improved cardiometabolic biomarkers (Muñoz‐Hernández et al. 2020). Markedly, the decline in insulin levels observed in human studies contrasts with the elevation reported in animal models, suggesting that enhanced peripheral insulin action may be a key mechanism in humans (Muñoz‐Hernández et al. 2020).

IF has gained attention for its potential therapeutic applications in improving insulin function and glucose homeostasis in both healthy individuals and those with type 2 diabetes (Furmli et al. 2018; Halberg et al. 2005; Varady 2016). These benefits arise through various mechanisms, including increased insulin‐mediated glucose uptake rates, improved glucose tolerance, and reduced insulin resistance. Moreover, the advantages of IF are reinforced by its ability to elevate plasma insulin levels and decrease the Homeostasis Model Assessment (HOMA) index in diabetic rats (Belkacemi et al. 2012).

A RCT comparing the effects of a 2‐day intermittent energy restriction (IER) regimen with continuous energy restriction (CER) in overweight or obese individuals revealed superior reductions in hepatic insulin resistance under IER (Harvie et al. 2011). The study demonstrated a 24% decline in the HOMA index under IER versus only 4% under CER (*p* = 0.001), with an additional 25% reduction observed after 2 energy‐restricted days. Notably, these improvements in insulin sensitivity occurred despite similar reductions in body fat between the two groups (−4.5 kg for IER vs. −3.6 kg for CER, *p* = 0.34) (Harvie et al. 2011).

Additionally, a study using a hyperinsulinemic euglycemic clamp reported improved insulin‐mediated whole‐body glucose uptake and inhibition of adipose tissue lipolysis following 2 days of normal eating post‐fasting (Halberg et al. 2005). The authors linked these improvements to increased adiponectin concentrations during the fasting period. Conversely, a separate study found no significant effects on peripheral glucose uptake or hepatic insulin sensitivity following a 2‐week IF intervention (Soeters et al. 2009).

To examine the impact of TRF on body weight, white adipose tissue, and metabolic biomarkers, a recent animal model experiment allocated two distinct cohorts of animals to receive either a standard or cafeteria diet, subsequently subjecting them to TRF and unrestricted food access. The investigation revealed that the TRF protocol enhanced body weight regulation and dyslipidemia mitigation in the cafeteria‐diet model while also stimulating the browning of white fat with thermogenic properties, potentially elucidating the anti‐obesity efficacy of TRF (Aouichat et al. 2020).

In humans, IF has been shown to have positive effects on insulin and other metabolic markers. According to human studies, 22 days of ADF promoted a notable 50% reduction in insulin secretion. Additionally, research comparing the impact of caloric restriction and the 5:2 diet over 4.5 months demonstrated greater declines in insulin levels and insulin resistance in the IF group despite significant weight loss in both groups (Heilbronn et al. 2004; Hoddy et al. 2020; Kim et al. 2021).

An RCT by Xie et al. (2022) involving humans evaluated the effects of early TRF over 5 weeks, reporting improved insulin sensitivity, decreased fasting plasma glucose, reduced body mass and adiposity, alleviated inflammation, and enhanced gut microbial diversity. In contrast, neither the midday TRF nor the control group exhibited comparable improvements. Nevertheless, no significant differences in blood pressure, circulating lipid concentrations, HbA1c, hsCRP, sleep quality, or appetite were observed between the three groups despite reductions in energy intake, indicating that differences in metabolic outcomes were not attributed to variations in caloric intake, suggesting the timing of food intake plays a distinct role in metabolic health (Xie et al. 2022).

### 3.2 Glucose Tolerance and Fasting Glucose

IF has also shown promising effects on glucose tolerance, particularly in individuals with early‐stage metabolic disorders. A 5‐week early TRF trial in individuals with prediabetes reported improved pancreatic beta‐cell responsiveness to glucose, as indicated by oral glucose tolerance test findings (Sutton et al. 2018). In contrast, a study investigating the effects of the 5:2 diet in subjects with type 2 diabetes over 12 weeks found an increased risk for hypoglycemia associated with this dietary pattern, highlighting potential safety concerns (Corley et al. 2018).

Evidence suggests that IF positively influences insulin sensitivity and glucose metabolism—particularly in individuals with dysregulated glucose and lipid profiles (Kim et al. 2021). Meta‐analyses of clinical trials have shown that IF reduces fasting blood glucose, glycosylated hemoglobin, insulin plasma levels, and HOMA‐IR while decreasing BMI, body weight, and waist circumference (Albosta and Bakke 2021; Haupt et al. 2021; Muñoz‐Hernández et al. 2020; Song and Kim 2023; Yuan et al. 2022). Additionally, IF has been associated with decreased fasting glucose levels, fasting insulin levels, insulin resistance, and leptin concentrations, alongside elevated adiponectin and HDL‐C levels, improved hepatic fat content, C‐peptide, and glucagon levels (Albosta and Bakke 2021; Haupt et al. 2021; Muñoz‐Hernández et al. 2020; Song and Kim 2023; Yuan et al. 2022).

Beyond its role in weight management, IF has exhibited potential in addressing insulin resistance and cardiometabolic parameters in patients with diabetes, though more research is needed to validate these findings. A comprehensive analysis by Herz et al. (2023) examined the interplay between fasting and diabetes, uncovering the potential of fasting to mitigate the risk of hypoglycemia in type 1 diabetes mellitus, reduce glycemic variability, and optimize lipid metabolism in both types of diabetes mellitus. Additionally, evidence has demonstrated that fasting can enhance insulin sensitivity, suppress endogenous glucose production in diabetes, promote weight loss, and refine body composition, reinforcing its potential as a therapeutic modality for managing type 1 and type 2 diabetes mellitus, it and can be judiciously implemented with professional oversight when deemed appropriate (Herz et al. 2023).

The proposed association between these alterations and IER suggests an impact on the autophagy–lysosomal pathway and the promotion of beta‐cell progenitor proliferation. Additionally, the observation of this pattern revealed stimulation in the reconfiguration of the gut microbiota (Muñoz‐Hernández et al. 2020). Building upon previously discussed findings, it is pertinent to compare the varying forms of IF and their respective impact on insulin and glucose regulation. A growing body of rigorous evidence suggests that, across both diabetic and non‐diabetic populations, the various forms of IF—namely ADF, TRF, and 5:2 fast—consistently attenuate fasting blood glucose, insulin, and HOMA‐IR, promoting glycemic stability and regulation (Xiaoyu et al. 2024; Cho et al. 2019; Nowosad and Sujka 2021; Schroor et al. 2023). Comparative analyses reveal no substantial variation in efficacy between regimens; however, SUCRA scores suggest 5:2 fast or twice‐weekly fasting for general health advantages beyond glycemic control (Xiaoyu et al. 2024). Furthermore, the effect of 5:2 fast extends further in type 2 diabetes, where it demonstrates a modest yet consistent advantage in glycemic management (Silva et al. 2023). Conversely, ADF appears marginally superior in enhancing insulin resistance relative to other forms of IF, though its overall advantage remains limited (Cho et al. 2019). While both ADF and TRE effectively lower glucose and insulin primarily via weight reduction, ADF appears to have a marginal advantage in attenuating metabolic outcomes (Silva et al. 2023). However, considering variations in study designs and participant characteristics is crucial, given their potential influence on the interpretation of results, despite the predominantly positive effects of IF on insulin sensitivity reported in most studies.

## 4 Thyroid Hormones and Energy Metabolism

### 4.1 Thyroid Hormone Modulation in Fasting State

Thyroid hormones, including triiodothyronine (T3) and thyroxine (T4), are critical for regulating metabolic rate, energy expenditure, and thermogenesis. The thyroid gland systematically releases T3 and T4, each distinguished by their iodine content, regulating numerous physiological functions essential for homeostasis (Pirahanchi et al. 2023). The hypothalamus–pituitary–thyroid (HPT) axis serves as a key system governing energy utilization. The hypothalamic thyrotropin‐releasing hormone (TRH) stimulates the release of thyroid‐stimulating hormone (TSH) from the anterior pituitary gland. Subsequently, TSH prompts the thyroid to discharge T4, which is subsequently transformed into active T3 by type I and II deiodinase enzymes (Pirahanchi et al. 2023). Understanding thyroid hormone modulation during fasting provides valuable insights into its relationship with circadian rhythms, a key factor in metabolic health.

### 4.2 Implications for Metabolic Rate

The influence of IF on the HPT axis has been assessed in both human participants and animal models. Fasting promotes notable reductions in TRH and TSH levels, with the subsequent impact on thyroid hormone levels. While both human participants and animal models report these reductions, animal models often exhibit more acute responses. Peripheral T3 levels tend to decrease under fasting conditions, supporting the hypothesis that HPT axis downregulation serves as an energy‐conserving mechanism in response to food scarcity, cold exposure, starvation, and infection (Karimi et al. 2024). As illustrated in Table 1, experimental and observational studies in animals and humans reveal significant fluctuations in thyroid hormones across different fasting protocols and durations. A human study found that serum T3 levels declined by 55% following a 24‐h fasting period, with no variation in TSH levels found after fasting (Merimee and Fineberg 1976). Moro et al. reported similar results, where T3 decreased significantly in the TRE group (−6%, *p* = 0.01) compared to an increase in their counterparts in the normal diet group (+9%, *p* = 0.07) (Moro et al. 2020). Similar trends were reported for ADF and 8‐h TRF (isocaloric, unrestricted eating within the specified time frame) protocols, with fasting reducing circulating T3 levels without affecting TSH levels in both short‐ and long‐term scenarios (Stekovic et al. 2019; Moro et al. 2016). Beyond hormonal fluctuations, IF decreases HPT axis activity, influencing appetite regulation and body weight, particularly in stressed animal models. The intricate molecular pathways underlying these changes in the HPT axis during fasting have been linked to leptin, TRH, TSH, and thyroid hormones (García‐Luna et al. 2023; Kim et al. 2021).

Several studies highlighted the potential connection between thyroid hormone regulation and insulin resistance (Martinez and Ortiz 2017; Azizi 2015). Research by Martinez and Ortiz in animals suggests fasting‐induced thyroid hormone alterations may contribute to insulin dysregulation, whereas Azizi's work on Islamic fasting underscores the distinct physiological changes occurring during this form of fasting (Martinez and Ortiz 2017; Azizi 2015). Mahadevan et al. (2017) further contribute to this discourse by demonstrating that TSH testing results can be influenced by food intake timing, highlighting the importance of accounting for fasting‐feeding status in thyroid function assessment. Additionally, Akasheh et al. compared the weight loss efficacy between ADF and daily calorie restriction in individuals with subclinical hypothyroidism and reported that both methods were equally effective without significantly altering thyroid hormone levels (Akasheh et al. 2019). Notably, fasting and postprandial states can affect thyroid function assessments, with TSH levels decreasing after food intake (Nair et al. 2014). These findings have implications for the diagnosis and management of hypothyroidism, particularly in subclinical hypothyroidism and during pregnancy. In male rats, both fasting and food restriction reduced serum T4, while fasting specifically decreased serum T3, influencing the expression of T3‐responsive genes (de Vries et al. 2014).

Furthermore, aside from studying IF in healthy individuals, the impact of IF in individuals with either established or early‐stage thyroid disorders remains unexplored. Limited evidence suggests potential benefits in autoimmune thyroid diseases, such as Hashimoto's disease. Persistent inflammation is a major factor in autoimmune conditions, and IF's anti‐inflammatory properties may offer therapeutic advantages. A review on Ramadan fasting reported a reduction in both fT4 and fT3 levels while increasing TSH among individuals with thyroid dysfunction using levothyroxine during fasting (Barati et al. 2023). Conversely, individuals with hypothyroidism exhibited a decrease in fT3, fT4, and TSH. Gender‐based variations were observed, with women and elderly individuals experiencing more prominent changes, whereas others reported greater susceptibility in men (Barati et al. 2023; Belal et al. 2023). Despite these findings, further research is needed to elucidate the efficacy and safety of IF in these contexts.

Understanding IF's long‐term effects on thyroid hormone regulation remains an ongoing process. The observed fluctuations in thyroid hormones during fasting appear to be temporary adaptations, functioning as energy‐conserving mechanisms rather than signs of dysfunction (Sui et al. 2024; García‐Luna et al. 2023). Most findings suggest that these changes are generally mild and reversible upon refeeding, especially with carbohydrates (Azizi 1978; Basolo et al. 2019). While human evidence remains moderate, conclusions are often derived from short‐term, controlled settings or extrapolated from animal models (Kim et al. 2021; Azizi 1978; Basolo et al. 2019; Varady et al. 2022). Long‐term effects are still unclear, warranting caution in individuals with pre‐existing thyroid disorders when considering IF as an intervention (Kim et al. 2021; Varady et al. 2022). Data on the safety and suitability of such a dietary approach requires careful assessment with proper dosing of medication when needed. Robust, large‐scale clinical trials are needed to clarify the clinical relevance of these findings.

## 5 Cortisol and Hypothalamic–Pituitary–Adrenal (HPA) Axis

### 5.1 Cortisol Response to Fasting

The influence of IF on glucocorticoid regulation and the HPA axis has been widely debated. The endocrine coordination mechanisms involving humoral and neural signals, particularly glucocorticoids such as cortisol and dehydroepiandrosterone (DHEA), are overseen by the biological clock in the hypothalamus (Marciniak et al. 2023; Jaschke and Wang 2023; Douglass et al. 2023). Fasting activates agouti‐related peptide neurons (AgRP) in the hypothalamus, which suppress corticotropin‐releasing hormone neurons (CRH), lowering GABAergic inputs (Jaschke and Wang 2023; Douglass et al. 2023). This pathway promptly initiates HPA axis signaling, leading to increased secretion of glucocorticoids, chiefly cortisol (Jaschke and Wang 2023; Douglass et al. 2023). A vital steroid hormone, cortisol, plays a crucial role in preserving the body's equilibrium by influencing diverse metabolic processes (Marciniak et al. 2023; Jaschke and Wang 2023; Douglass et al. 2023). Excessive stimulation of the HPA axis and heightened levels of glucocorticoids can influence conditions associated with obesity (Marciniak et al. 2023). Additionally, visceral adipose tissue is implicated in cortisol regulation, particularly in response to fasting and acute stress (Marciniak et al. 2023). Fasting‐driven activation of the HPA axis has been linked to metabolic enhancement and potential lifespan extension in animal models (Marciniak et al. 2023). Investigations into the modulation of adrenal cortical steroids during fasting provide crucial insights into circadian rhythm alterations associated with fasting. Furthermore, energy balance and caloric intake appear to modulate the endocrine response to fasting, emphasizing the interplay between nutrient availability and hormonal regulation.

### 5.2 Dehydroepiandrosterone and Cortisol Balance

A review exploring fasting and cortisol dynamics revealed that IF intensifies cortisol secretory bursts, which may hold implications for metabolic homeostasis through the cortisol‐to‐DHEA ratio (Balsevich et al. 2019; Chawla et al. 2021; Kim et al. 2021; Marciniak et al. 2023; González‐Bono et al. 2002). Glucocorticoids—vital effector molecules of the HPA axis—play a pivotal role in appetite modulation, where stress inhibits food consumption through cortisol actions (Balsevich et al. 2019; Chawla et al. 2021; Kim et al. 2021; Marciniak et al. 2023). Extended fasting has been shown to rapidly increase cortisol levels, with 5 days of fasting resulting in peak hormonal alterations (Balsevich et al. 2019; Chawla et al. 2021; Kim et al. 2021; Marciniak et al. 2023). Interestingly, TRE strategies elicit distinct effects on cortisol secretion. Nonrestrictive TRE lowers cortisol levels, whereas isocaloric TRE raises them, highlighting the importance of food timing and composition on cortisol secretion (Balsevich et al. 2019; Chawla et al. 2021; Kim et al. 2021; Marciniak et al. 2023).

Fasting‐induced endocrine modulations that extended beyond glucocorticoid regulation. Studies indicate that a 72‐h fast elevates cortisol, adrenocorticotropic hormone (ACTH), adrenaline, and noradrenaline, while simultaneously decreasing the TSH and T3 (Beer et al. 1989), as summarized in Table 1. Similarly, Fichter and Pirke demonstrated that weight loss and caloric restriction influence the HPA axis, leading to elevated plasma cortisol levels (Fichter and Pirke 1986). Palmblad et al. observed similar effects, with total energy withdrawal inducing changes in growth hormone, cortisol, and thyroid hormone concentrations (Palmblad et al. 1977). Moreover, Komaki et al. reported fasting‐induced immune system changes, particularly in lymphocyte subsets and natural killer cell activity, potentially mediated by adrenal hormone modulation (Komaki et al. 1997).

Various factors influence the regulation of cortisol levels in the body. Korbonits et al. (1996) found that food intake elevates cortisol levels, particularly in obese individuals. Additionally, Tabata et al. (1991) observed that low blood glucose levels during exercise can trigger cortisol secretion. Further analysis by Bergendahl et al. (2000) highlighted the impact of age on cortisol responses, where older individuals experience a greater increase in cortisol levels during fasting. Lastly, Tegelman et al. (1986) documented that controlled fasting increases cortisol secretion while influencing other hormone levels. Taken together, the findings indicate that fasting‐induced cortisol secretion facilitates energy mobilization, aiding metabolic adjustments to food deprivation as a part of an adaptive survival response (Jaschke and Wang 2023; Douglass et al. 2023; Djordjevic et al. 2008). Additionally, cortisol levels appear to be sensitive to variables such as nutrient intake, glycemic status, age, and fasting duration. Considering the scarcity of clinical trials and research on conditions such as adrenal insufficiency and Cushing syndrome, challenges remain. In light of current evidence, fasting causes hyperstimulation of the HPA axis, resulting in mild cortisol elevations (Marciniak et al. 2023; Jaschke and Wang 2023; Douglass et al. 2023). However, the clinical relevance of these findings in humans remains unclear, as most of the mechanistic pathways are derived from animal models (Marciniak et al. 2023), which—although valuable for understanding physiological pathways—have limited direct translation of these results to humans. While fasting appears to enhance metabolic adaptability (Jaschke and Wang 2023; Douglass et al. 2023; Djordjevic et al. 2008), available data largely pertain to acute short‐term effects with minimal understanding of chronic long‐term adaptations. Consequently, there is a critical need for well‐designed, high‐quality human trials to delve into the enduring effects of IF on hormonal balance, ensuring its safe clinical application.

## 6 Reproductive Hormones and Fertility

### 6.1 Impact on Male Reproductive Hormones

IF has been linked to alterations in testosterone and luteinizing hormone (LH) in men, particularly during extended fasting periods. Interestingly, fasting may be perceived as a physiological stressor by females, suggesting a nuanced relationship between metabolic states and reproductive health (Sadowska et al. 2022).

Studies on rodents indicate mixed effects on reproductive hormones. One study reported that fasting increased estradiol levels and significantly decreased LH levels (Kumar and Kaur 2013). Similarly, a 30‐week ADF increased the likelihood of irregular or absent estrous cycles, whereas a 22‐week TRF plan with regular chow consumption had minimal impact on estradiol levels and instead demonstrated improvements in reproductive function (Hua et al. 2020). Further investigations across species reinforce the variable impact of fasting on reproductive hormones and behaviors. In rabbits, fasting decreased the LH peak and 17‐β‐estradiol levels, correlating with reduced receptivity and fertility rates (Brecchia et al. 2006). Conversely, ewes subjected to fasting exhibited decreased insulin and insulin‐like growth factor 1 (IGF‐1) levels alongside increased progesterone levels (Kiyma et al. 2004). Meanwhile, zebra finches undergoing short‐term fasting displayed higher corticosterone levels, decreased testosterone concentrations, and reduced reproductive behaviors (Lynn et al. 2010). In young rats, fasting suppressed the hypothalamic–hypophysial–gonadal axis, negatively impacting reproductive function (Kumar and Kaur 2013). Among *Salvelinus alpinus* , longer periods of fasting lowered reproductive output (Frantzen et al. 2004), underscoring species‐specific hormonal adaptations.

A review of human trials by Cienfuegos et al. indicated that IF modulates androgenic indicators, with reductions in testosterone and the free androgen index (FAI) observed in both genders (Cienfuegos et al. 2022). However, sex hormone‐binding globulin (SHBG) levels may increase in premenopausal females, suggesting potential compensatory mechanisms. IF, particularly in the form of time‐restricted eating, has been associated with significant testosterone declines, most notably in active, fit individuals. Despite this decline, studies consistently reported no impairment in muscular development or performance (Moro et al. 2016, 2020, 2021; Stratton et al. 2020). TRE alone or in combination with resistance exercise led to significant reductions in testosterone levels—from a 3.12% decrease at 2 months (*p* = 0.01) and a 16.81% reduction at 12 months (*p* = 0.0004) in the TRF group compared to a marginal impact in the control group (MORO et al. 2021) with a maximal reduction of 27% (*p* = 0.006) (Moro et al. 2020). However, an 8‐week TRE intervention did not compromise physical outcomes in non‐elite, physically active individuals engaged in resistance training (Stratton et al. 2020); in fact, it improved the peak power output‐to‐body weight ratio (Moro et al. 2020), demonstrating that muscle performance metrics remain preserved despite the shift in anabolic androgens.

Moreover, short‐term fasting influences the reproductive axis in both men and women. One study demonstrated that a 48‐h fast in men showed a decrease in LH, follicle‐stimulating hormone (FSH), and testosterone concentrations, as well as a decrease in LH pulse frequency (Cameron et al. 1991). However, a recent systematic review by Kalsekar et al. (2024) yielded mixed findings, reporting significant reductions in total testosterone and FAI, yet no significant changes in LH and FSH. In contrast, IF resulted in a significant increase in IGF‐1 and SHBG levels (Kalsekar et al. 2024). Notably, the review indicated that IF had substantial effects on various hormonal parameters, with decreases in the levels of FSH, LH, estradiol, prolactin, dehydroepiandrosterone sulfate, and anti‐Müllerian hormone, alongside significant increases in TSH and SHBG levels (Kalsekar et al. 2024).

Furthermore, a 10‐day total fast in men decreased serum FSH and serum testosterone concentrations (Klibanski et al. 1981). Similarly, a 3‐day fast in women decreased the number of LH pulses while maintaining follicle development or cycle lengths (Olson et al. 1995). In women, fasting does not compromise reproductive function but does advance the central circadian clock (Berga et al. 2001). In contrast to men, women's reproductive axis appears more resilient to short‐term fasting, with no major alterations observed following a 2.5‐day fast (Bergendahl et al. 1999). IF protocols such as Ramadan fasting have been evaluated for their effects on reproductive function and the HPT axis, with one study finding no significant alterations in male reproductive function or the HPT axis (Azizi 2015).

Emerging evidence highlights sex‐specific differences in fasting responses. As seen in Table 1, a 48‐h fasting trial reported analogous glucose intolerance in males and females, yet significant decreases in 17‐β‐estradiol among females (Solianik et al. 2023 ). Moreover, fasting activated the sympathetic nervous system in both sexes; however, a more pronounced stress‐related response, marked by an increase in norepinephrine and psychological stress, was noted in females (Solianik et al. 2023 ). This sexual dimorphism in stress perception and estradiol fluctuations may underscore the enhanced insulin response and insulin resistance frequently observed in females relative to males.

In addition, a study investigating carbohydrate supplementation during fasting in men found that urinary gonadotropin excretion increased, which may be a potential mechanism for the decline in serum testosterone levels, suggesting direct testicular suppression rather than a secondary regulatory effect (Kyung et al. 1985). This highlights the complex interplay between nutrition, hormonal regulation, and reproductive health.

### 6.2 Impact on Female Reproductive Hormones

In women—particularly those with obesity or polycystic ovary syndrome (PCOS)—IF notably time‐restricted eating has been shown to improve androgenic markers (Cienfuegos et al. 2022; Velissariou et al. 2025). TRE significantly reduced total testosterone (*p* = 0.048) and FAI (*p* = 0.001), while markedly increasing SHBG (*p* < 0.001), potentially enhancing eumenorrhea and fecundity (Li et al. 2021). An 8‐h TRE protocol resulted in a 2% reduction in body weight, with more pronounced effects observed when dietary patterns favor caloric intake earlier in the day—suggesting benefits for females with PCOS via improved insulin sensitivity and androgen regulation (Harvie et al. 2010; Li et al. 2021; Jakubowicz et al. 2013). A RCT further explored the impact of meal timing in lean females with PCOS. Participants with early intake of calorie‐dense meals exhibited a 50% drop in free testosterone and a rise exceeding 100% in SHBG, paralleled with improved ovulatory responses and metabolic parameters (Jakubowicz et al. 2013). This is contrary to the group with late intake of calorie‐dense meals, where an elevation in estradiol (E2) was observed (Jakubowicz et al. 2013). Elevated E2 beyond normal levels is known to impair the endocrine axis regulating female fertility.

Current evidence regarding IF and sex hormones is heterogeneous in quality, with many human studies constrained by small sample sizes or a narrow population focus (e.g., females with PCOS), limiting generalizability. Conversely, animal studies offer mechanistic insights, particularly related to differences in responses to IF between both sexes, yet their direct translation is narrow (Kumar and Kaur 2013; De Basea et al. 2024; Rius‐Bonet et al. 2024). Findings suggest that IF may lower androgen markers in both sexes, potentially alleviating hyperandrogenism in females while raising concerns in men as the full effects are not clear due to possible adverse effects on metabolic regulation and reproductive behavior, despite improvements in general biomarkers and physical performance in trained males. Additionally, fasting may induce unintended reduced caloric intake (Cienfuegos et al. 2020) that may impair the anabolic environment necessary for muscle maintenance, which requires further investigation concerning physical health. Notably, IF has been shown to improve SHBG levels in females, consequently reducing androgen bioavailability. In contrast, no significant changes in SHBG were observed in males, raising questions about the hormonal regulation in this group. The evidence indicates a decrease in total testosterone in some studies without the anticipated increase in SHBG (Moro et al. 2016, 2020, 2021; Stratton et al. 2020), suggesting different regulatory mechanisms at play in males. Collectively, these findings advocate for further research on the role of IF—especially eTRE and its long‐term effects on reproductive hormones in both sexes—to fully understand the safety and efficacy of this approach as a potential complementary therapy for reproductive health disorders.

## 7 General Health Outcomes of IF

### 7.1 Chronometabolic Role of Melatonin

Melatonin, often referred to as the “hormone of darkness,” is primarily secreted by the pineal gland in response to the onset of darkness, orchestrating the body's internal clock with the external light–dark cycle. This hormone modulates sleep–wake patterns and other circadian rhythms, influencing metabolic homeostasis.

Fasting protocols, including Ramadan fasting and short‐term IF, have been shown to alter the timing and amplitude of melatonin secretion (Chawla et al. 2021). Studies indicate changes in the circadian distribution of hormones and physiological variables during Ramadan fasting, particularly affecting body temperature, cortisol, melatonin, and glycemia (Bogdan et al. 2001; Roky et al. 2004). Diurnal IF during Ramadan decreases melatonin, ghrelin, and leptin levels in overweight and obese subjects while maintaining circadian integrity (Al‐Rawi et al. 2020). However, lower melatonin levels at 22:00 h were observed during fasting (Almeneessier et al. 2017). Animal studies further demonstrate that melatonin rhythms in mice can shift in response to the timing of food availability, with nighttime feeding resulting in rhythms similar to those observed during ad libitum feeding while considering the nocturnal nature of mice (Froy et al. 2009). In humans, a 3‐day fast advanced the peak of melatonin secretion without shifting its circadian rhythm, reinforcing that melatonin timing is predominantly governed by the light–dark cycle rather than the feeding pattern (Berga et al. 2001; Chawla et al. 2021; Kim et al. 2021). Table 2 summarizes the interactions between melatonin and various fasting protocols, along with their metabolic impact.

**TABLE 2:** General health outcomes of intermittent fasting.

Hormone category	Fasting type	Type of participants		Principal findings	References
		Humans	Animals		
Melatonin and circadian rhythm	Ramadan Fasting	Humans	/	↑ Cortisol, ↓ Melatonin, ↓ Testosterone, (−) fT4, (−) fT3, ↑ TSH, (−) LH, ↓ FSH, ↑ Prolactin, ↓ GH	Bogdan et al. (2001)
		Humans		↓ Amplitude of body temperature, ↑ Cortisol, ↓ Melatonin, ↓ Glycemia, ↑ Uric acid, ↑ Urea, ↑ HDL, ↑ Apoprotein A1, ↓ LDL	Roky et al. (2004)
		Overweight and Obese		(−) Cortisol, ↓ Ghrelin, ↓ Melatonin, ↓ Leptin	Al‐Rawi et al. (2020)
		Humans		↓ Melatonin	Almeneessier et al. (2017), Faris et al. (2020)
	3‐day Fast	Humans		Advancement in peak secretion without affecting circadian rhythm	Berga et al. (2001)
Adiponectin and metabolic outcomes	TRF	Humans	/	↑ Adiponectin significantly, ↑ Insulin sensitivity, ↑ HDL‐C, ↓ TG	Zhang et al. (2022), Moro et al. (2020)
				↑ Adiponectin, ↓ BMI, ↓ WC, ↑ HDL‐C, ↓ HOMA‐B, ↓ IR	Karras et al. (2021), Moro et al. (2016)
	ADF	/	Mice	↑ Adiponectin, ↓ Visceral fat	Varady et al. (2010)
				↓ Body weight in 100% ER, ↓ Adipocyte size in 100% ER, 50% ER, ↑ Adipose tissue TG metabolism in 100% ER, 50% ER, ↑ Adiponectin in 50% ER, ↑ Free Fatty Acids	Varady et al. (2007)
	Ramadan Fasting	Humans	/	↓ Body weight, ↓ BMI, ↓ Glucose, ↓ Insulin, ↑ Insulin sensitivity, ↓ IR, ↓ Adiponectin	Gnanou et al. (2015)
				(−) Adiponectin, ↓ Leptin	Gaeini et al. (2021)
				↑ Adiponectin, ↑ FBG, ↑ TG, ↓ BMI, (−) TNF‐α, ↑ Insulin Sensitivity, ↓ Body fat %	Feizollahzadeh et al. (2014)
	72‐h fast	Humans	/	↓ Glucose, ↓ Insulin, ↓ Leptin, (−) Adiponectin	Merl et al. (2005)

Abbreviations: −, no change; ADF, alternate‐day fasting; BMI, Body mass index; ER, energy restriction; FBG, fasting blood glucose; FSH, follicle‐stimulating hormone; fT3, free triiodothyronine; fT4, free thyroxine 4; GH, growth hormone; HDL, high‐density lipoprotein; HOMA‐B, homeostasis model assessment of β‐cell function; IR, insulin resistance; LDL, low‐density lipoprotein; LH, luteinizing hormone; TG, triglycerides; TNF‐α, tumor necrosis factor α; TRF, time‐restricted feeding; TSH, thyroid‐stimulating hormone; WC, waist circumference.

Studies suggest that melatonin may regulate insulin secretion and glucose metabolism by interacting with melatonin receptors MT1 and MT2. Heightened melatonin levels, particularly during glycemic challenges such as meal consumption, have been shown to hinder insulin release and impair glucose tolerance (Chawla et al. 2021). These effects are thought to be mediated by a mutated allele for melatonin receptors expressed in insulin‐producing pancreatic islets, notably the B1 (MTNR1B) receptor. Individuals carrying this allele are at increased risk for hyperglycemia and type 2 diabetes. Consequently, abstaining from meals for 2–3 h before bedtime and for an hour upon rising, when melatonin levels are elevated, may be beneficial (Chawla et al. 2021; Garaulet et al. 2020).

Emerging evidence suggests that aligning meal timing with elevated melatonin levels may negatively affect metabolic health. For instance, a randomized crossover trial demonstrated that consuming meals 1–2 h before bedtime, when melatonin levels are typically elevated, impaired insulin sensitivity, glucose tolerance, and fasting plasma glucose levels in participants (Garaulet et al. 2022). Moreover, dawn‐to‐sunset fasting TRF has been associated with beneficial outcomes in obesity, metabolic syndrome, and nonalcoholic fatty liver disease (Mindikoglu et al. 2017). A 5‐h meal delay demonstrated shifts in plasma glucose and adipose tissue PER2 mRNA rhythms, further supporting the metabolic effects of circadian‐aligned feeding (Wehrens et al. 2017). Haouari et al. further support these findings by observing variations in caloric consumption, body weight, and circadian cortisol evolution in response to the inverted food intake rhythm during Ramadan fasting (Haouari et al. 2008).

These findings provide important consideration for Ramadan fasting, where higher melatonin levels during the circadian night suppress insulin responses to meals, leading to irregular glycemic responses. Ramadan fasting studies have reported reduced melatonin secretion, potentially serving as a compensatory response to fasting‐induced insulin regulation disruptions (Chawla et al. 2021; Garaulet et al. 2020). However, this hypothesis requires further research for confirmation.

Despite evidence linking melatonin to insulin dynamics and metabolic adaptations, the precise molecular mechanisms remain incompletely understood. Further studies should focus on how melatonin regulates pancreatic insulin release and the clinical implications of optimizing meal timing with circadian rhythms.

### 7.2 Adiponectin and Metabolic Outcomes

Adiponectin, an adipose‐derived hormone, plays a fundamental role in modulating diverse facets of metabolic health by activating signaling pathways such as AMPK, which controls glucose uptake and fatty acid oxidation in the skeletal muscle and liver (Nguyen 2020). Beyond its metabolic functions, adiponectin suppresses pro‐inflammatory cytokine production, including tumor necrosis factor‐alpha (TNF‐alpha) and interleukin‐6 (IL‐6), thereby mitigating inflammation and enhancing insulin sensitivity (Nguyen 2020).

Given its key role in glucometabolic regulation, adiponectin is widely studied for its role in developing metabolic syndrome. Its ability to reduce pro‐inflammatory cytokines, oxidative stress, and improve insulin resistance while exhibiting anti‐atherosclerotic and beta‐cells protective effects highlights its potential for disease prevention (Faris et al. 2020). The increased adiponectin levels following IF have significant clinical implications for managing metabolic syndrome and associated disorders (Faris et al. 2020). Table 2 details the key findings regarding IF‐induced alterations in adiponectin circadian rhythms and its effects on metabolic and anthropometric parameters.

Several studies have assessed the effects of IF on adiponectin regulation, yet findings remain inconsistent. Stable adiponectin levels were observed during a 72‐h fast in normal‐weight and overweight individuals (Merl et al. 2005). Similarly, modified ADF in mice influenced adipocyte size and lipid metabolism but did not significantly alter adiponectin levels (Varady et al. 2007). In contrast, ADF raised adiponectin levels, comparable to the effects of daily energy restriction (Varady et al. 2010). Further studies indicate fasting‐associated improvements in insulin resistance and metabolic stress, reinforcing the role of adiponectin in metabolic adaptation (Feizollahzadeh et al. 2014). On the other hand, divergent outcomes were also reported in religious fasting studies; adiponectin levels decreased during Ramadan fasting (Gnanou et al. 2015) but increased with Orthodox fasting (Karras et al. 2021). Fasting patterns such as TRF and modified fasting regimens have been shown to contribute to weight loss and improved metabolic parameters (El Khatib and Yassin 2019). Moro et al. found that early TRF demonstrated a tendency to elevate serum adiponectin levels by 33% (*p* = 0.08), though not significantly (Moro et al. 2020). The increase achieved significance after accounting for fat mass (50%, *p* = 0.02), compared to a marginal insignificant increase in the normal diet group (5%) (Moro et al. 2020). Similarly, Zhang et al. reported in a RCT that participants following early and late TRF exhibited significant increases in adiponectin levels compared to those following late TRF (Zhang et al. 2022).

While IF may modestly increase serum adiponectin, its effects in acute and short‐term fasting appear inconsistent. These discrepancies underscore the need for careful interpretation. Further long‐term studies are needed to define its metabolic significance.

## 8 Cancer Prevention: Fasting and Hormonal Regulation in Cancer Therapy

The fasting process induces changes in various metabolic pathways as the body transitions to a state in which energy and metabolites are derived from adipose tissue. This adaptive shift protects normal cells from the effects of chemotherapeutic treatments, thereby reducing cellular differentiation and metabolic activity, while paradoxically increasing the susceptibility of cancer cells to treatment as they defy the growth‐inhibiting signals enforced by fasting conditions (Tiwari et al. 2022).

Mounting evidence suggests a complex and intertwined relationship between cancer, the endocrine system, circadian rhythm regulation, and IF. Disruptions in circadian regulations are increasingly linked to heightened cancer risk and progression, particularly in hormone‐sensitive malignancies such as breast, ovarian, and prostate cancers (Savvidis et al. 2024; Miro et al. 2023; Hadadi and Acloque 2021). The circadian clock orchestrates essential hormonal and metabolic processes, and its dysregulation—as in the case with modern lifestyle or shift work—may induce hormonal imbalance, immune suppression, and cell cycle disruptions, thereby providing a favorable microenvironment for tumorigenesis (Savvidis et al. 2024; Miro et al. 2023; Hadadi and Acloque 2021).

Key metabolic pathways are tightly linked to cancer development, including insulin signaling, insulin‐like growth factor‐1 (IGF‐1), and mTOR. These pathways activate PI3K/Akt/mTOR signaling, which promotes cell division and survival, reprogramming metabolism in line with the Warburg effect observed in various tumors (Kasprzak 2020; Lau and Leung 2012; Floyd et al. 2007). In ovarian and prostate cancers, activation of these pathways contributes to reducing cell‐to‐cell adhesion by inhibiting proteins such as E‐cadherin, facilitating tumor invasion and metastasis (Lau and Leung 2012; Siech et al. 2021). Notably, disruption of these pathways—particularly mTOR—is common in approximately 70% of tumors, modulating growth, nutrient influx, and maintaining energy sufficiency to sustain anabolic activity, along with impairing autophagy, thereby augmenting oncogenic potential (Mir et al. 2023; Floyd et al. 2007). In turn, circadian misalignment adversely affects these pathways, promoting carcinogenic activity (Savvidis et al. 2024; Miro et al. 2023; Hadadi and Acloque 2021). Conversely, IF—especially when synchronized with circadian rhythm (i.e., TRF)—may mitigate cancer risk by attenuating insulin/IGF‐1/mTOR signaling, eliciting an anticancer proteomic profile without imposing severe energy deficit, enhancing metabolic homeostasis by reducing glucose availability, IGF‐1, and insulin; reducing TNF‐alpha and IL‐1‐beta; and restoring circadian integrity as indicated in several gene expression analyses (Salvadori et al. 2021; Tiwari et al. 2022; Godos et al. 2025). The clinical findings suggest that early‐day TRF and prolonged nocturnal fasting are associated with cancer risk modulation by enforcing circadian stability and mitigating inflammation (Godos et al. 2025; D'cunha et al. 2022). As Table 3 illustrates, animal studies have demonstrated the potential of IF to delay cancer growth and improve survival rates, mitigate the risk of specific cancer types, and manipulate the energy metabolism of tumor cells, thereby impeding their growth while enhancing antitumor immune responses (Buschemeyer et al. 2010; Giannakou et al. 2020; Zhao et al. 2021). Thereby recalibrating cellular pathways and attenuating oxidative stress, collectively inhibiting tumor progression (Mir et al. 2023; Floyd et al. 2007).

**TABLE 3:** Intermittent fasting effects on cancer outcomes and treatment.

Hormone category	Fasting type	Type of participants		Principal findings	References
		Humans	Animals		
Cancer response	TRF + PA	Humans	/	↓ Pro‐inflammatory cytokines, ↓ TNF‐alpha. ↓ IL1‐beta, ↓ IGF1, ↓ Insulin, ↓ Glucose, ↓ Leptin, ↓ IGF‐1 and mTOR pathways	Salvadori et al. (2021), Tiwari et al. (2022)
	IF + CR	Humans	/	(−) Cancer growth, (−) Survival rate, ↓ IGF‐1 in the group fasted 2 day/week and the group with 28% CR 7 days/week. ↓ Fat mass and lean body mass in the group with 28% CR 7 days/week	Buschemeyer et al. (2010)
		/	Animals	↓ Cancer incidence, ↓ Number of lesions, ↓ Inflammation of the tissues, ↓ Risk of certain types of cancer	Giannakou et al. (2020)
	IF + Chemotherapy	Humans	/	↑ Treatment efficacy, ↓ Side effects, ↑ Patient outcomes	Gabel et al. (2021)
	IF	Humans		↑ Autophagy, ↑ Immune response, ↑ Tumor‐infiltrating lymphocytes, ↑ Bone marrow lymphoid progenitor cells, ↑ Antioxidant defenses pathways, ↓ Inflammation, ↓ Oxidative stress	Psara et al. (2023)

Abbreviations: −, no change; TRF, time restricted feeding; PA, physical activity; TNF‐α, tumor necrosis factor α; IL‐1β, interlukin‐1β; IGF‐1, insulin like growth factor‐1; mTOR, mammalian target of rapamycin; CR, calorie restriction; IF, intermittent fasting.

When combined with chemotherapy, IF has been found to enhance treatment efficacy, reduce side effects, and improve patients' health outcomes (Gabel et al. 2021). Studies suggest that IF could potentially reduce chemotherapy‐related toxicity and tumor growth (Clifton et al. 2021; de Gruil et al. 2022). Moreover, IF has been associated with various health‐promoting effects in cancer patients, including autophagy induction, immune response augmentation, and oxidative stress attenuation (Psara et al. 2023). Fasting and fasting‐mimicking diets can modulate growth factors and metabolite levels, potentially decreasing cancer cell survival and enhancing treatment outcomes (Nencioni et al. 2018). Fasting before and during chemotherapy may augment tumor cell response to treatment, potentially curtail tumor progression, and enhance chemotherapy effectiveness (Sadeghian et al. 2021). Similarly, Brandhorst and Longo (2016) indicated that diverse forms of reduced caloric intake, such as periodic fasting, fasting‐mimicking diets, and dietary restriction, can confer various advantageous effects in cancer prevention and treatment. These interventions have been shown to induce cellular protection, amplify the efficacy of cancer therapies, and hinder the adaptability and survival capabilities of cancer cells (Nencioni et al. 2018; Raffaghello et al. 2008; Tinkum et al. 2015). Both Lee and Longo (2011) and Safdie et al. (2012) further reinforce the potential of fasting in reducing chemotherapy side effects and toxicity, advocating for its integration into oncological protocols. However, dietary restriction—when prolonged—may prove less effective than fasting and can lead to chronic weight loss (Lee and Longo 2011).

While preliminary evidence indicates potential benefits for symptom management and reduced treatment toxicities, several challenges hinder the introduction of IF into clinical practice for cancer patients. For instance, patient compliance with fasting regimens may be challenging, particularly for individuals undergoing intensive chemotherapy or experiencing cancer‐related cachexia and malnutrition. The overall body of evidence demonstrates promising potential, though its quality varies. While animal and molecular studies provide robust mechanistic insights, clinical findings on targeted cellular pathways remain inconsistent, often due to pathway redundancy and compensatory mechanisms. However, rigorous, large‐scale clinical trials are necessary to establish safety, efficacy, and optimal protocols for integrating fasting and circadian biology into cancer care, considering individual endocrine and metabolic profiles, which may offer new avenues for personalized therapeutic options, facilitated by interdisciplinary collaborations among oncologists, clinical dietitians, and researchers.

## 9 Metabolic Memory and the Challenge of Sustainability

Many dietary interventions have shown significant benefits in improving metabolic and endocrine functions in both animal and human studies. However, a major hurdle in nutritional science is the temporary nature of these improvements; metabolic parameters often revert to baseline after the dietary regimen ends. IF, particularly associated with Ramadan, can induce adaptive endocrine reprogramming, leading to significant changes in the gut microbiome, such as increased abundance of certain bacterial families (e.g., Lachnospiraceae and Ruminococcaceae), which are associated with improved metabolic health and reduced inflammation (Su et al. 2021). However, these changes—along with weight loss and improved glucose metabolism—tend to resolve after the fast ends, underscoring the need for sustained interventions to maintain health gains. A recent meta‐analysis reported that IF leads to significant improvements in glycemic and anthropometric markers compared to other dietary interventions in the study in individuals with type‐2 diabetes, but these improvements are reversible after cessation. It also revealed long‐term benefits of continuous fasting, with more sustained effects on HbA1c and body weight observed compared to shorter periods (Liu et al. 2025). In order to prolong these benefits, strategies such as periodically reintroducing fasting protocols, supporting a diverse microbiome, and focusing on meal timing even after the official fast ends may help maintain positive shifts in metabolic health and reduce the risk of chronic diseases (Su et al. 2021).

## 10 Conclusions and Future Perspectives

In summary, IF represents a promising dietary approach with systemic effects on circadian alignment, endocrine modulation, and metabolic regulation. Its influence on hormonal rhythms and gut microbiota composition offers potential benefits in maintaining metabolic homeostasis and attenuating oncogenic risk. Current evidence, largely derived from preclinical models, is constrained by heterogeneous methodologies, short intervention durations, and limited sample sizes, in addition to targeting specific populations, hindering the translation of such findings into consistent, clinically meaningful outcomes in humans. These gaps are especially critical when considering implementations in vulnerable populations with endocrine disorders such as type 1 and 2 diabetes or with tumors, where metabolic demands and treatment contexts introduce additional layers of complexity. To advance therapeutic applications, future research should prioritize longitudinal, well‐controlled, large‐scale human trials to validate these findings and establish their relevance within therapeutic frameworks tailored to oncology and endocrine care and consider perspectives from both genders. Integrating this paradigm into clinical practice holds the potential to transform nutritional science into a dynamic tool for precision medicine.

## Author Contributions

**Wijdan Shkorfu:** conceptualization (lead), writing – original draft (lead). **Abdulmannan Fadel:** conceptualization (equal), investigation (equal), supervision (lead), writing – review and editing (lead). **Mohammed Hamsho:** validation (equal). **Yazan Ranneh:** conceptualization (equal), supervision (equal). **Hafiz Muhammad Shahbaz:** visualization (supporting).

## Conflicts of Interest

The authors declare no conflicts of interest.

## Data Availability

Data sharing not applicable to this article as no datasets were generated or analyzed during the current study.
